# Spatio-Temporal Features of Visual Exploration in Unilaterally Brain-Damaged Subjects with or without Neglect: Results from a Touchscreen Test

**DOI:** 10.1371/journal.pone.0031511

**Published:** 2012-02-08

**Authors:** Marco Rabuffetti, Elisabetta Farina, Margherita Alberoni, Daniele Pellegatta, Ildebrando Appollonio, Paola Affanni, Marco Forni, Maurizio Ferrarin

**Affiliations:** 1 Fondazione Don Carlo Gnocchi ONLUS, Falconara Marittima, Ancona, Italy; 2 Clinica Neurologica, Ospedale San Gerardo, Monza, Italy; 3 Dipartimento di Neuroscienze, Università di Milano Bicocca, Milan, Italy; University of Bologna, Italy

## Abstract

Cognitive assessment in a clinical setting is generally made by pencil-and-paper tests, while computer-based tests enable the measurement and the extraction of additional performance indexes. Previous studies have demonstrated that in a research context exploration deficits occur also in patients without evidence of unilateral neglect at pencil-and-paper tests. The objective of this study is to apply a touchscreen-based cancellation test, feasible also in a clinical context, to large groups of control subjects and unilaterally brain-damaged patients, with and without unilateral spatial neglect (USN), in order to assess disturbances of the exploratory skills. A computerized cancellation test on a touchscreen interface was used for assessing the performance of 119 neurologically unimpaired control subjects and 193 patients with unilateral right or left hemispheric brain damage, either with or without USN. A set of performance indexes were defined including Latency, Proximity, Crossings and their spatial lateral gradients, and Preferred Search Direction. Classic outcome scores were computed as well. Results show statistically significant differences among groups (assumed p<0.05). Right-brain-damaged patients with USN were significantly slower (median latency per detected item was 1.18 s) and less efficient (about 13 search-path crossings) in the search than controls (median latency 0.64 s; about 3 crossings). Their preferred search direction (53.6% downward, 36.7% leftward) was different from the one in control patients (88.2% downward, 2.1% leftward). Right-brain-damaged patients without USN showed a significantly abnormal behavior (median latency 0.84 s, about 5 crossings, 83.3% downward and 9.1% leftward direction) situated half way between controls and right-brain-damaged patients with USN. Left-brain-damaged patients without USN were significantly slower and less efficient than controls (latency 1.19 s, about 7 crossings), preserving a normal preferred search direction (93.7% downward). Therefore, the proposed touchscreen-based assessment had evidenced disorders in spatial exploration also in patients without clinically diagnosed USN.

## Introduction

Unilateral spatial neglect (USN) is a neuropsychological disorder whereby brain-damaged patients fail to report events occurring on the space side that is usually contralateral to the side of a unilateral lesion. These patients also fail to explore that space side. The deficit is more frequent and severe after damage to the right hemisphere, typically involving the left-hand side of space [1]–[3].

Some of the most used tests for the diagnosis of USN are cancellation tests where patients are asked to cancel out targets arranged in a display located in front of the participant, with the centre of the display being usually aligned with the mid-sagittal plane of the participant's body. Targets may or may not be interspersed with distracters. These tests include line [4], circle [5], letter [6], star [7], bell [8] and symbol [9] cancellation.

These pencil-and-paper tests readily provide scores such as the number of crossed out/omitted targets in the left- and right half-space of the test display, the total exploration time, and allow the computation of the average time per target. These scores are computed after the participants terminate the task. The on-line recording of exploration strategies requires a close monitoring of the patients, possibly by more than one examiner.

According to these approaches, the patient classification on an on-off basis is determined by the comparison of the actual score, generally computed as the difference between omitted targets on the left and omitted targets on the right half-space with cut-off values. This threshold-based criterion is congruent with the simple assumption of neglect as a “failure” and therefore works when a patient “fails”. Nonetheless, it has been observed that pencil-and-paper test classification may be inadequate to detect mild USN when an individual fulfils a neglect test by obtaining a normal score. This observation is valid even though the way in which the task is accomplished is different than that of a normal performance [10]. Moreover, it has been observed that the behavioral assessment of USN in daily life is often more sensitive than pencil-and-paper tests [11].

The complexity of a USN deficit, even when not detected by standard clinical tests, has been nonetheless taken into consideration [12]. Specific ad-hoc tests and protocols, providing not only scores but also indexes related to the temporal and spatial aspects of the performance, have been proposed in order to assess the manifold aspects of neglect and particularly the interplay with cognitive domains such as memory [13]–[14], attention [15], motor control and intention [16]–[17], and sensory system [18]–[19].

A survey on chronometrical approaches [20] shows that an increasing search time possibly occurs in any decay of searching performance. Chronometrical studies generally involved patient-operated switches or keys [21]–[27] however other technical solutions have been adopted, including touchscreen [28].

Other works focused on the spatial features of search tasks. It is relevant to note that this approach can be partly supported by the classic testing: pencil-and-paper cancellation tests allow for an off-line analysis evidencing for instance the lateral position of omitted targets. Indeed, several papers reported counting of omissions per vertical strip sectors, computing local scores about omission/detection ratios [18], [29]–[31] and evidencing that, at least in some right-hemisphere-damaged patients, the more leftward the position of the stimulus is, the worse the local score will be.

These latter approaches, which only look at omission/detection scores, require that the patients show a USN (namely, he/she omits targets) but do not directly show “how” the space is explored. Without adopting specific technologies, Samuelsson recorded the search patterns verbally reported by patients [32]. A research team from the University of Kent proposed a setup based on a digitizing tablet [33]–[35]. Mapstone [36], adopting a setup with eye-trackers, studied the spatial distribution of eye fixation in a visual search paradigm finding that even in the absence of clinically observable USN, subjects with right unilateral brain lesions show altered behavior on the contralesional hemi-space. Similar findings have been reported by studies performed using an eye tracking device in USN patients [37]–[38]. Karnath analyzed exploration of a large space by eye and head movements adopting magnetic transducers placed on the eye and on the head [39]. Parton proposed an exploration task on a touchscreen for studying the re-exploration of already touched items which may be alternatively simply tagged, cancelled or enhanced [40].

Mark and Woods adopted a video recording of a cancellation test in order to identify the searching path. Therefore, they quantified distances between the successively detected stimuli and the occurrence of crossings along the search path. They computed an index based on the largest correlation coefficient between stimuli coordinates and sequential order, evidencing the presence of an organized search modality [41]–[42]. They studied the strategies of spatial exploration in patients with mild or undetectable USN. Based on their observations, they concluded that USN and the patterns of organization of visual exploration are not strictly related, even though an association is often observed. Such results were substantially confirmed by Manly [43]. He proposed a detailed analysis of the performance of control subjects and right-brain-damaged patients with USN in a Star cancellation test whose measure is obtained from an a-posteriori analysis of video recordings of the tests. They focused on indexes either referred to temporal and/or spatial features, observing significant slowing when cancelling targets towards left and significant decrease of search organization, while observing that those aspects do not correlate with classic neglect scores.

In conclusion, despite the fact that clinical diagnosis of USN in exploratory tasks mainly relies on the omission of contralesional targets, the latter studies reported, that are just a sample from the literature, evidence of altered spatial and temporal features of the search patterns in both USN patients and in patients without apparent clinical evidence of the disorder; i.e. subjects who do not show target omission in conventional tests. These patients may be considered to suffer from a milder impairment of visual-explorative and attentive cognitive functions and are still of interest in the clinical management in order to pursue a full functional recovery. This represents a strong rationale in the development of testing methods capable of evidencing such mild disturbances and, at the same time, compatible with a clinical context.

The present paper is based on a methodological innovation concerning the implementation of a computerized cancellation test adopting a touchscreen interface [44]–[45] that functions as the so-called *tablet*. This approach, which is characterized by an extremely user-friendly interface, allows defining numerical indexes related to “how” the exploration task has been carried out. Particularly it has been shown how it is possible to assess a relation between performance indexes and laterality (where unilateral spatial neglect can be considered the extreme deficit in task involving exploration along a lateral direction), making it possible to explore even mild disturbances when moving attention towards the affected side. The objectives of the present paper are to further improve the analysis of task performance as measured by the touchscreen apparatus by adding some new indexes, and to report the results obtained from large groups of patients with unilateral brain damage, either with or without clinical evidence of unilateral spatial neglect, and control normal subjects.

## Materials and Methods

All experimental activities have been approved by the Health Research Agency of the Italian Ministry of Health and by authors' institutional review board (Ethics Committee of the Fondazione Don Carlo Gnocchi ONLUS). Informed consent has been obtained from all the participants about the investigation conducted according to the principles expressed in the Declaration of Helsinki.

### Participants

The participants included in this multi-centre study were 119 neurologically unimpaired controls recruited among the staff (not directly related to this research) and visitors of the Don Gnocchi hospitals and among those attending recreational centers for elderly of the city of Milan. One-hundred-ninety-eight with unilateral hemispheric damage to the left or the right hemisphere were recruited for the study group from the rehabilitation hospitals of the Don Carlo Gnocchi Foundation in Milano, Parma and Sarzana, and in the San Gerardo Hospital, Monza, Italy. Inclusion criteria were a neurological diagnosis of focal unilateral brain damage, due to a vascular accident, a neoplastic disease or a head injury, an educational level of five or more years of schooling, and a normal or corrected-to normal vision. Exclusion criteria were clinical evidence and/or history of alcohol/drug abuse, psychiatric disorders, non-cooperative behavior, and inability to perform a run-in test (at least one target detected among 4 targets and 4 distracters). All patients included (in particular, no patient failed the run-in test) were given a preliminary screening using the Mini Mental State Examination [46]–[47] and a pencil-and-paper neuropsychological battery for USN, including a line cancellation test [4], a letter cancellation test [6], [48], the Wundt-Jastrow area illusion test [49], a sentence reading test [50] and a line bisection test [7]. Using this screening battery, patients with right or left hemispheric damage were subdivided into two groups: patients without USN (USN−, namely patients whose performance was within the normal range in all tasks), and patients with USN (USN+, namely patients who showed USN in one or more of the pencil-and-paper tests included in the screening battery). Finally, subjects not able to detect at least 10 out of 40 targets were excluded from the study because the indexes computation produces unreliable values when a limited number of cancellations is available. This final exclusion step was applied to only 5 patients with right brain damage and severe neglect at the clinical assessment. Table 1 summarizes the demographic and neurological features of control subjects (CONTROL) and patients. Apparently, no patient with left brain lesion evidenced USN, consequently only three groups of patients were identified and coded RUSN− for right lesion and no neglect, RUSN+ for right lesion and neglect, LUSN− for left lesion and no neglect. Lesions were localized by CT or MRI Scan; Table 1 also reports the number of patients showing a lesion in a cerebral lobe or a subcortical damage with lesions involving more than one lobe in some patients. Patients' groups resulted balanced (as evidenced by ANOVA analyses) according to education and time elapsed since brain lesion, while age and performances in MMSE test evidenced differences among groups despite the large overlap of those indexes' values.

**Table 1:** Subjects enrolled in the study.

	Control	LUSN−	RUSN−	RUSN+
**N (Female/Male)**	119 (86/33)	72 (31/41)	66 (29/37)	55 (23/32)
**Age (mean+/−std)**	58.9+/−15.2	60.6+/−14.2	56.1+/−15.8	64.7+/−14.5
**School years (mean+/−std)**	11.4+/−4.1	9.5+/−4.5	9.6+/−4.3	8.6+/−4.0
**Handedness (Right/Left/missing)**	114/5/0	61/9/2	61/4/1	45/10/0
**Lesion etiology (Vascular/Traumatic/Neoplastic/missing)**		56/9/3/4	44/11/6/5	48/3/3/1
**Months since lesion (1–3/4–12/>12/missing)**		26/12/25/9	24/12/22/8	28/12/13/2
**Temporal lesion**		23	18	20
**Frontal lesion**		22	27	17
**Parietal lesion**		28	23	16
**Subcortical lesion**		22	19	14
**Occipital lesion**		5	6	6
**Lesion site not reported**		7	8	12
**MMSE**		23.4±8.7	27.9±2.1	26.0±4.0
**Line cancellation**	Cutoff ≥1	0.0±0.2	0.0±0.1	1.1±2.4
**Letter cancellation**	Cutoff ≥3	0.1±1.4	0.1±0.7	11.6±12.6
**Wundt–Jastrow test**	Cutoff ≥2	0.0±0.6	0.0±0.2	2.0±17.3
**Sentence reading**	Cutoff ≥1	0.4±1.5	0.0±0.0	1.3±2.0
**Line bisection**	Cutoff ≤7	8.4±1.8	8.8±0.4	5.1±2.3

Demographic data, brain lesion etiology (the time elapsed from lesion is referred to patients with vascular or traumatic etiology only) and anatomical location, and neuropsychological profile of the subjects enrolled in the study.

### Experimental setup and global

**Figure 1:**
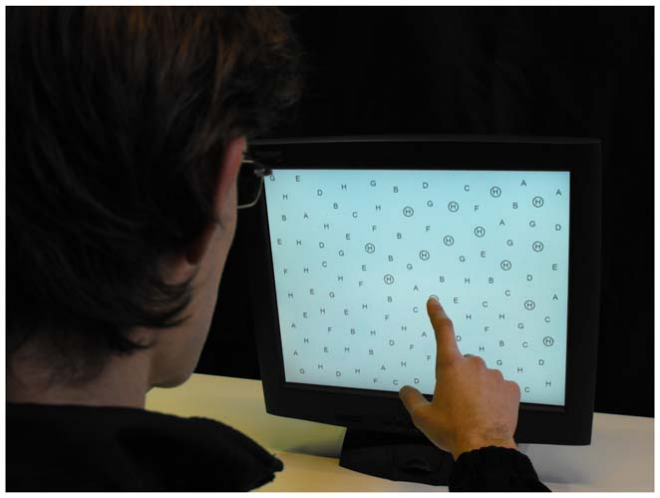
Experimental setup with subject touching target displayed by a touchscreen monitor.

The detailed hardware and software features of the experimental setup have previously been described [44] and are summarized here. A 19″ touchscreen monitor displayed a uniform distribution of 120 stimuli, either letters or shapes, including 40 targets and 80 distracters. Each participant was seated in front of the screen with the mid-sagittal plane of the trunk aligned with the centre of the screen. The task consisted of touching with the index finger all the targets that the subject was able to detect. The real-time testing software excluded any sliding behavior and only allowed a touch-and-go behavior for touching an item. No effect or recording was associated with a finger touch on the blank space among the stimuli. Once the target touched, it would be circled (see Figure 1). Each participant performed two tests in random order, one on the letter distribution and one on the shapes distribution. Control subjects used their dominant hand, while patients used hands of the ipsilesional unaffected side. Once the participant declared having completed the task, the test software stored the performance's raw outcome on files. No time limit was imposed but if the participant had not declared the test conclusion after 10 minutes, he/she was asked whether the test was finished.

### Global scores

The experimental raw outcome consisted of a time series including, for each touched item (including perseverated touches), the time T of occurrence of the event and the X (rightward axis) and Y (downward axis) screen coordinates of each touched item.

The following global scores were computed

- NS, unilateral Neglect Score, defined as the difference between the number of targets cancelled on the left and on the right sides of the screen, expressed as a percentage of the total number of targets (for brain-damaged patients, it is considered the difference between the targets cancelled on the contralesional side and on the ipsilesional side);
- NT, Number of touched Targets;
- ND, Number of touched Distracters;
- NP, Number of Perseverations, i.e., number of repeated aware touches on targets which have been already touched once.

### Mathematical models and indexes of test performance

**Figure 2:**
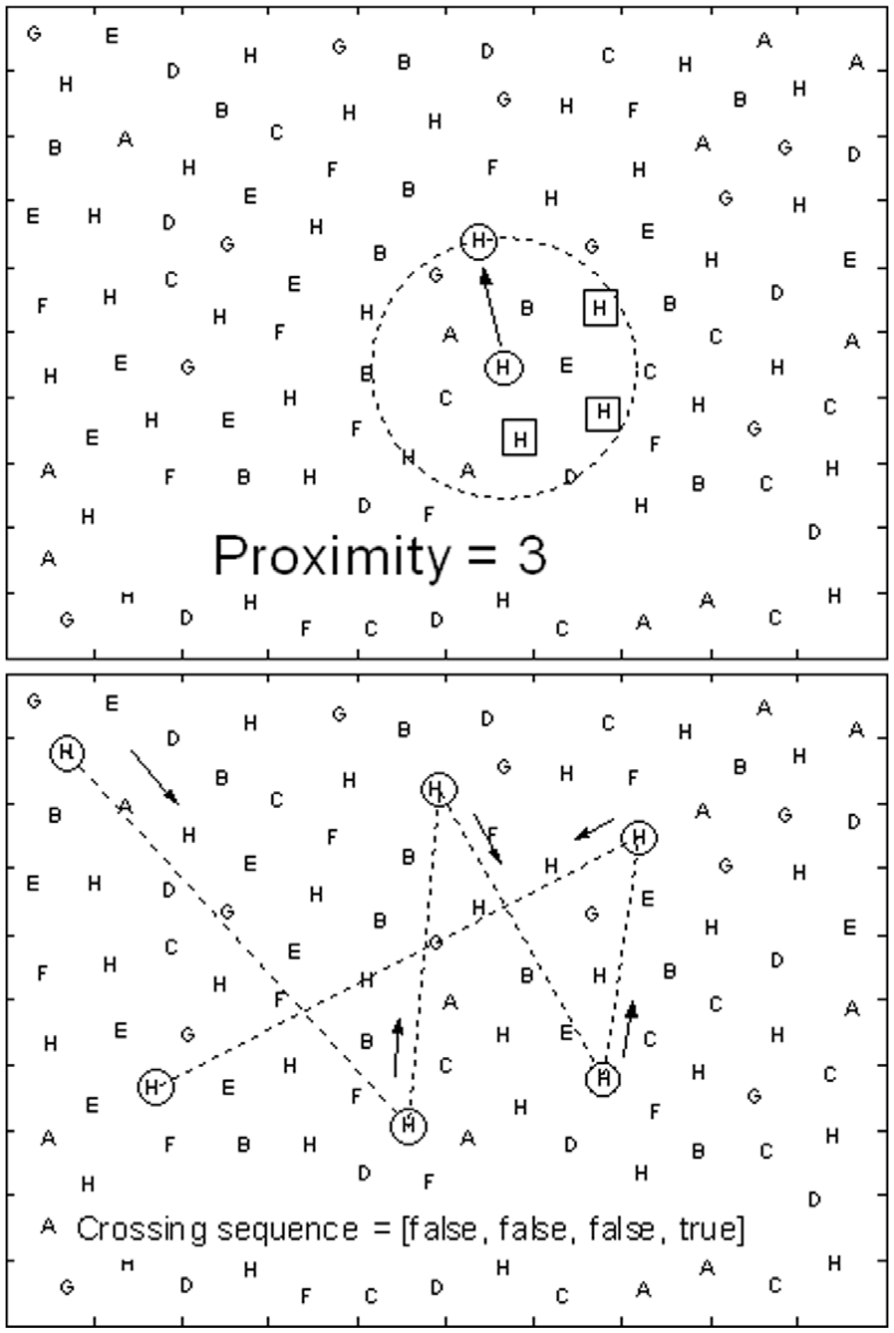
Examples of the computation of Proximity (2a) and Crossing (2b) variables.

Given the time series T_i_, X_i_, Y_i_ where i = 1: n, n being the total number of touched items, a set of variables were defined:

- *latency*, array of real numbers, being the time in seconds between each target detection and the previous detection, defined as L_i_ = T_i_−T_i−1_;
- *distance*, array of real numbers, being the distance between the currently detected target and the previous one, expressed in fractions of screen width, defined as D_i_ = |([X_i_ Y_i_]−[X_i−1_ Y_i−1_])|
- *search speed*, array of real numbers, defined as S_i_ = D_i_/L_i_;
- *proximity*, a vector of integer numbers, defining P_i_ as the number of not yet cancelled targets that were closer to the previously touched item (i−1) than the actual i-th one (see figure 2a);
- *crossing*, array of Boolean variables, defined as true if any crossing of previous search path occurred, otherwise false (see figure 2b).

Those variables allow for the definition of an extended set of numerical indexes which are related to global aspects of the test performance.

The participant's performance is described by the following global indexes:

- *LI*, *Latency Index*, defined as the median value of the Latency variable;
- *DI*, *Distance Index*, defined as the median value of the Distance variable;
- *SI*, *Search Speed Index*, defined as the median value of the SearchSpeed variable;
- *PI*, *Proximity Index*, defined as the mean value of the Proximity variable;
- *NC*, *Crossing Index*, defined as the number of occurred crossings divided by the total number of touched items;
- *LP*, *Longest Path*, defined as the maximum number of consecutive touches without crossing (max length sequence of false crossing).

A second set of indexes was defined in order to quantify a relation between any performance index and the lateral coordinate. This was relevant to check whether a more leftward position was related to some significant changes in target detection.

The elective mathematical tool was the regression analysis of a generic variable vs. the X lateral coordinate, assumed as the independent factor [44]. The numerical value of the identified gradient, which is the slope of the fitting line, can also be assumed as the difference of the values that the index displayed on the extreme right and on the extreme left (e.g., a latency gradient of −1.2 seconds means that an item took 1.2 seconds longer in order to be detected if it was located on the extreme left instead of the extreme right of the testing display). In order to relate the gradient to the side of the brain lesion, for the brain-damaged groups the gradient was referred to the contralesional/ipsilesional sides (in simple words, this required a sign change of the X-gradient for left brain-damaged patients only).

The following gradient indexes were considered:

- *LG, Latency Lateral Gradient*
- *DG, Distance Lateral Gradient*
- *SG, SearchSpeed Lateral Gradient*
- *PG, Proximity Lateral Gradient.*

**Figure 3:**
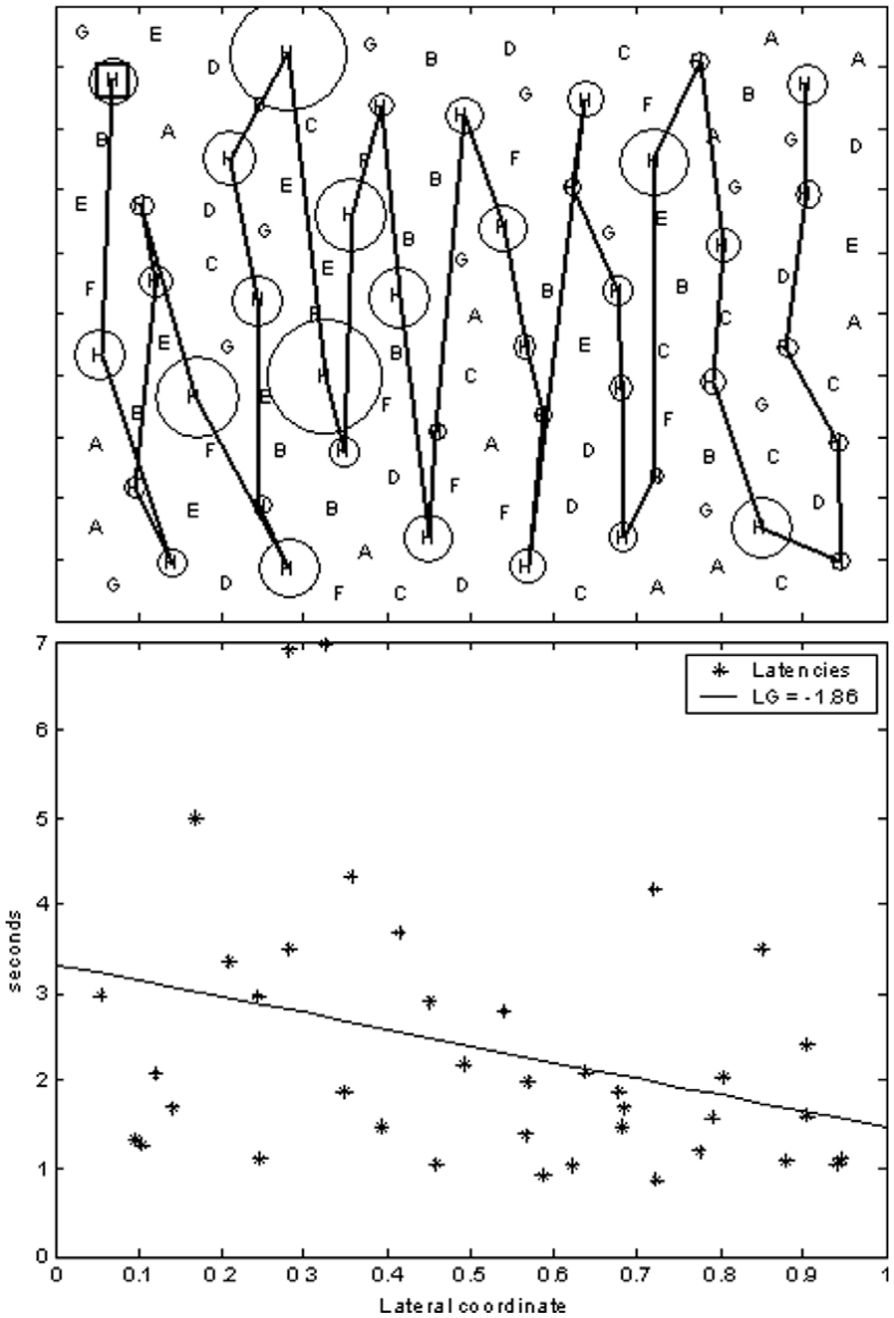
Example of Latency associated to performance. Given the occurred search path (3a), the Latency values related to each target cancellation are plotted versus the respective lateral coordinate (3b). The diameters of circles on targets (3a) quantify the specific cancellation latencies; the slope of the fitted regression line (3b) represents the Latency Gradient Index.

A graphical example of the computation of a gradient index is reported in figure 3.

A third set of indexes defined explores the hypothesis of a linear relation between time and one other variable; i.e. if an index increases (or decreases) constantly with time, the cross correlation analysis is the mathematical tool which provides the correlation coefficient r. The values of r that can be obtained range from 1.0, a perfect direct linear relation, to −1.0, a perfect inverse linear relation; the r null value, or close to zero, marks the relative independency of the two variables.

Given the time (t) and any variable (v), as N-long series of numbers, the formula to compute the r correlation coefficient r is
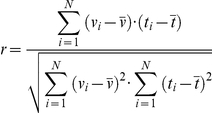



In the present study, considering the series of X, Y and T values, large absolute values of r may identify search patterns left-to-right or reverse, and top-down or reverse. The following direction indexes were used:

- *XC*, *Lateral Direction Index* (positive for rightward direction)
- *YC*, *Vertical Direction Index* (positive for downward direction).

**Figure 4:**
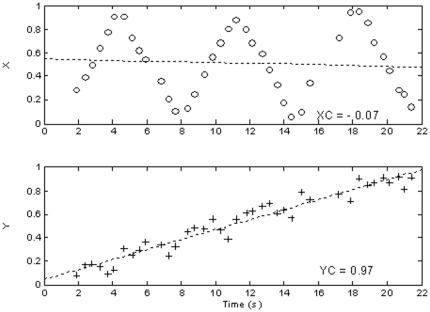
Example of XC and YC associated to performance. Given the occurred search path (4a), the horizontal (4b, top) and vertical (4b, bottom) coordinates of cancelled target are plotted vs. the time of their cancellation. The correlation coefficients XC and YC, respectively, provide evidence of the possible occurrence of a strong linear dependency between cancelled target locations and time: in the example shown the large positive YC coefficient evidences a downward search path.

A sample test is presented in figure 4 reporting the search path and timings which allow for the plotting of X and Y coordinates of the touches versus the timing of their occurrences. The best fit regression lines are superimposed and the correlation coefficients are reported as legends. The example shows an apparent upward strategy identified by the strong linear relation between Y and T.

Differences among groups were analyzed by Kruskall-Wallis non-parametric analyses of variance, with multiple comparisons when appropriate [51].

All mathematical computations and statistics were performed by Matlab (The Mathworks, USA) and Statistica (Statsoft, USA).

## Results

In table 2 the indexes distribution of the four experimental groups are summarized.

**Table 2:** Summary of experimental results.

Score/Index	Control (N trials = 238)	LUSN− (N trials = 144)	RUSN− (N trials = 132)	RUSN+ (N trials = 110)
**NS** (Neglect Score)	0.00 (−5.00< = >2.50)	0.00 (−2.50< = >5.00)	0.00 (−2.50< = >10.00)	7.50 (−2.50< = >37.50)
**NT** (N Targets)	40 (38< = >40)	40 (36< = >40)	40 (34< = >40)	34.50 (13< = >40)
**ND** (N Distracters)	0 (0< = >2)	1 (0< = >14)	0 (0< = >3)	1 (0< = >12)
**NP** (N Perseverations)	0 (0< = >1)	0 (0< = >4)	0 (0< = >2)	0 (0< = >5)
**LI** (Latency Index)	0.64 (0.44< = >1.15)	1.19 (0.61< = >2.37)	0.84 (0.49< = >1.87)	1.18 (0.61< = >3.07)
**LG** (Latency Gradient)	−0.02 (−0.64< = >0.55)	0.05 (−1.43< = >1.47)	−0.13 (−1.93< = >0.83)	−0.40 (−7.18< = >1.33)
**DI** (Distance Index)	0.15 (0.13< = >0.23	0.15 (0.13< = >0.25)	0.15 (0.13< = >0.19)	0.15 (0.13< = >0.23)
**DG** (Distance Gradient)	0.00 (−0.21< = >0.12	0.03 (−0.09< = >0.42)	−0.01 (−0.28< = >0.11)	0.00 (−0.22< = >0.16)
**SI** (Speed Index)	0.25 (0.15< = >0.35)	0.15 (0.08< = >0.25)	0.18 (0.09< = >0.31)	0.14 (0.07< = >0.24)
**SG** (Speed Gradient)	−0.01 (−0.15< = >0.09	0.02 (−0.16< = >0.18)	0.00 (−0.12< = >0.12)	0.02 (−0.19< = >0.23)
**PI** (Proximity Index)	1.82 (0.82< = >5.18)	2.53 (0.95< = >6.00)	2.14 (0.85< = >4.03)	2.42 (0.81< = >5.69)
**PG** (Proximity Gradient)	−0.51 (−7.51< = >3.62)	1.08 (−2.59< = >11.54)	−0.50 (−7.12< = >3.26)	0.69 (−7.52< = >11.85)
**NC** (N Crossings)	2.56 (0.00< = >16.22)	7.06 (0.00< = >30.88)	5.20 (0.00< = >20.51)	13.48 (0.00< = >42.86)
**LP** (Longest path)	30 (14< = >39)	23 (10< = >40)	26 (11< = >39)	16 (6< = >37)

A summary of the median values and, in brackets, the 5^th^ and 95^th^ percentiles of the defined indexes in the experimental trials (two for each participant) of the four subjects' groups. The across-group Kruskall-Wallis analysis of variance was significant (p<0.01) for all listed indexes except for the Distance Index DI (p = 0.41).

In order to identify the redundancies in the set of defined indexes occurring when two or more indexes are strictly related, and eventually to reduce the number of variables to be further analyzed, a data reduction approach by means of cross-correlation analysis (when 2 variables sufficiently correlate, this approach allows us to drop one of them) was adopted and applied to the global indexes (LI, DI, SI, PI, NC, LP). In our study, we fixed a threshold value for the correlation coefficient Pearson r of 0.7 for identifying a strong relation [52]. The data reduction analysis lets DI (r_PI,DI_ = 0.83), SI (r_LI,SI_ = −0.76) and LP (r_NC,LP_ = −0.74) indexes (and, therefore, the related, when computed, gradient indexes) to be discarded because of their strong association with LI, PI and NC indexes. While the justifications for keeping LI and NC were based on the their comparability with previously defined indexes and because of the clarity of their definition, PI was preferred to DI because only the former one showed a significant difference among groups (see caption of table 2). Therefore, the considered indexes set after the data reduction step included the scores NS, NT, ND, NP, the performance indexes LI, LG, PI, PG, NC and the direction indexes XC and YC.

**Figure 5:**
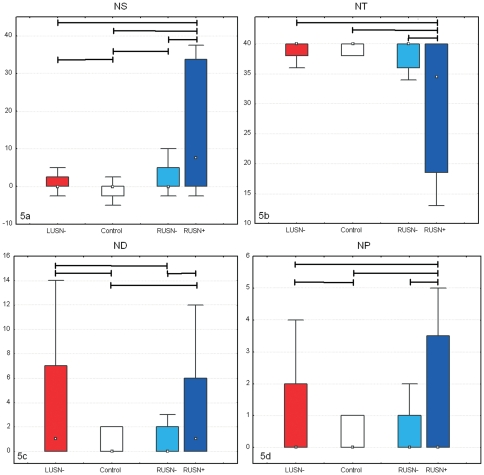
Result box plots (reporting median, quartiles and extreme values) of scores NS (5a, neglect score), NT (5b, target cancelled), ND (5c, distracters cancelled), and NP (5d, perseverations). Kruskall-Wallis ANOVA test is always positive, across-group statistically significant differences (post-hoc analysis, p<0.05) are marked by horizontal segments.

**Figure 6:**
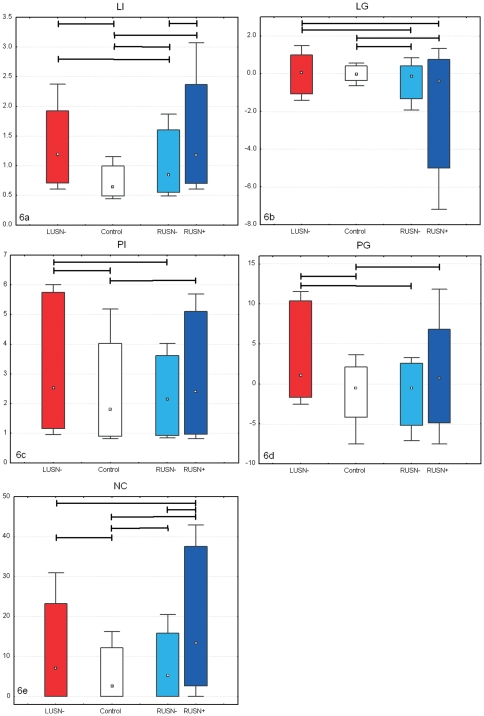
Result box plots (reporting median, quartiles and extreme values) of indexes LI (6a, Latency), LG (6b, Latency Gradient), PI (6c, Proximity), PG (6d, Proximity Gradient), and NC (6e, Crossings). Kruskall-Wallis ANOVA test is always positive, across-group statistically significant differences (post-hoc analysis, p<0.05) are marked by horizontal segments.

Control subjects evidenced performances whose global scores (NS, ND, NT, NP) were close to ceiling/floor values characterizing a “best” performance (i.e. all targets detected, no distracter cancelled, no perseverations observed) and, particularly, the neglect score NS was substantially null (see figure 5). The performance indexes (LI, LG, PI, PG, NC) expressed the following normal pattern (figure 6): generally, it took less than one second for each target detection (median LI was 0.64 s), between 2 subsequentially touched targets there was at least one closer target to the initial touched one that was undetected (median PI was 1.8, 95^th^ percentile was about 5) and rarely search path crossings were observed (median NC accounted for 2 to 3 crossing in the search for 40 targets with a maximum of 15 crossings). As no USN was shown by neurologically unimpaired subjects, even gradient indexes concerning latencies (LG) and proximities (PG) did not show any unbalance between right and left, and their values included the null value as a central one.

LUSN− patients compared to controls showed, as a group, a significant, though very small, neglect score (NS, it is worth noting that its median value was null) with an overall normal rate of target detection (NT) and significantly larger numbers of touched distracters (ND, median is 1 and may rise up to more than 10) and perseverations (NP, though median value is 0). These patients were slower (median LI is 1.19) and less efficient in search strategy (median PI is 2.53, NC is more than 7) than controls, occasionally showing laterally related effect (median PG is 1.1 with an overall bias for positive values). In general all the reported differences were due to outlier performances, while the differences between the median values of the global scores were either null (NS, NT, NP) or very small (ND).

RUSN− patients compared to controls showed a significant USN score (positive bias for NS, though median value is 0) particularly due to occurring extreme values; the other scores (NT, ND, NP) showed normal values and only few individuals presented themselves with abnormal values. Apparently, equal median values of the four global scores were observed in controls and RUSN− groups. The RUSN− group was slower (median LI is 0.84 seconds), with increasing slowness in the contralesional side of space (median LG is −0.13) and less efficient in the search strategy (median NC is more than 5 and can rise up to about 20) than controls.

RUSN+ patients compared to controls exhibited abnormal scores (NS, NT, ND, NP): they were slower (median LI is 1.21 seconds), with increasing slowness towards the left (median LG is −0.4, and may range down to −7) and they were less efficient in their search strategy (PI is 2.5; NC is 15) while proximity tends to decrease in the contralesional hemifield (median PG is 0.7, ranging from −7 to 11).

When comparing right brain-damaged patients, RUSN− exhibited a statistically significant intermediate behavior between RUSN+ and controls concerning neglect score (NS), latency (LI) and search efficiency (NC), while the extreme values of latency gradient in RUSN− were less severe than the ones showed by RUSN+.

If we compare left to right brain-damaged patients, LUSN− group showed lower values concerning distracters (ND), greater values concerning proximity (PI) and values in between RUSN− and RUSN+ for both latency and search efficiency (LI and NC).

**Figure 7:**
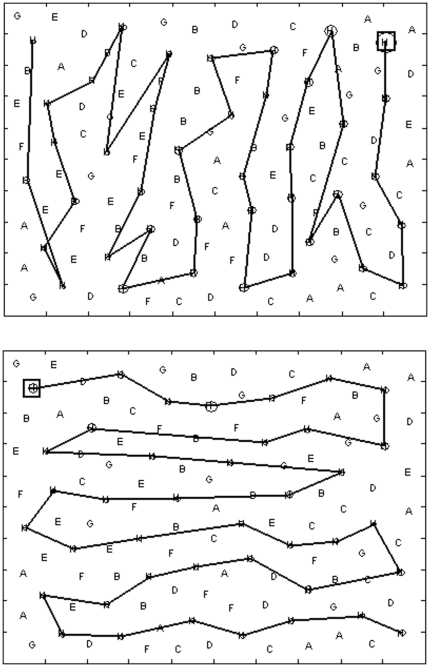
Example of polar plots of XC and YC associated to Search Direction. Given the occurred search paths, a polar plot of the related couple XC and YC provides a graphical representation of the Search Direction: example (7a) evidences a leftward Search Direction commonly observed in RUSN patients, conversely example (7b) shows a downward Search Direction often observed in controls.

**Figure 8:**
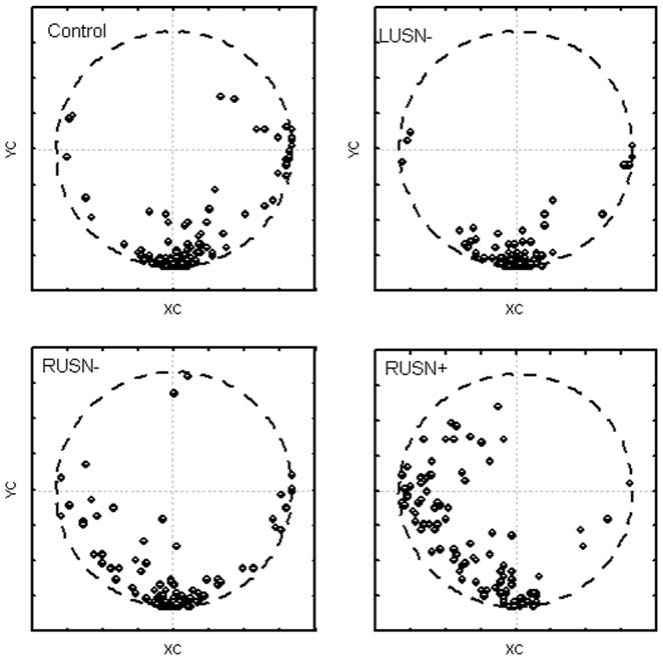
Result polar plots of all the couples (XC, YC) observed individually in the four groups: controls and LUSN− patients show a prevalence of a downward Search Direction, while RUSN patients show a tendency to orient leftward their Search Direction (more evident in RUSN+ patients). Arrows connecting the points to the origin are not plotted for clarity.

As for the searching path descriptors, the couple composed by the direction indexes XC and YC quantifies the search strategy. The vector connecting the origin of a XY reference frame to the point with coordinates (XC,YC) provides an immediate representation of a preferred search direction (the direction of the vector) and the strength of this search direction (the module of the vector). Examples about some paradigmatic search strategies are shown in figure 7, while a presentation of all the observed direction indexes for each group is presented in figure 8. When counting the preferred search direction according to 4 classes (UP/RIGHT/DOWN/LEFT according to the corresponding four 90° circular sectors), the results are summarized in table 3. A Chi-square test evidenced that the preferred search direction of control subjects is statistically different from a neutral figure characterized by a 25% of occurrence for each search direction, with a prevalence of a downward exploration organized in horizontal rasters. Moreover, the LUSN− group was not statistically different from the control group, while RUSN− showed a significant shift from the downward normal search strategy towards a left-oriented strategy organized in vertical rasters. Finally the RUSN+ subjects showed an even more apparent shift towards leftward oriented exploration, significantly more relevant with respect to control and RUSN− groups.

**Table 3:** Preferred Search Direction.

Group	N	DOWN	RIGHT	LEFT	UP
Control	238	88.3	9.2	2.1	0.4
LUSN−	144	93.7	4.2	2.1	0.0
RUSN−	132	83.3	6.1	9.1	1.5
RUSN+	110	53.6	3.6	36.4	6.4
Neutral reference		25.0	25.0	25.0	25.0

Observed frequencies in percentual of the Preferred Search Direction in all groups and a neutral reference figure in which all alternative Search Direction have the same weight. Statistical comparison (χ^2^ test, p<0.05) evidenced significant differences between control and the neutral reference, between RUSN− and control, between RUSN+ and control. LUSN− group was not different from controls. RUSN+ group was different from RUSN− group.

Given the observed differences among patients' groups, a further analysis has been carried out in order to verify whether those differences may be related to other factors, and particularly to those factors which showed significant differences among groups, namely age and MMSE outcome. The data from the three groups of patients was pooled and a correlation analysis between the two potential confounding factors and the test Performance Indexes was performed. The resulting correlation coefficients (previously considered in the data reduction stage when above 0.70) never exceeded 0.30 in the module (the largest coefficient being −0.27 for correlation between MMSE and ND, number of touched distracters) and generally was below 0.10, thus excluding an effect of the two potentially confounding factors on any of the touchscreen test outcomes.

Finally, as for the comparison of the pencil-and-paper letter cancellation test and the corresponding touchscreen-based test in the group of cases with right hemispheric damage, the following figure was obtained: both tests were negative in 66 subjects, positive in 32 subjects and 23 subjects showed discrepancy between the two testings. Among the latter group, there were 7 positive outcomes only in the pencil-and-paper test battery and 16 only in the touchscreen test. Though the result indicated a slightly better sensitivity of the touchscreen-based test, such difference was classified as not significant (p = 0.22; χ^2^ test).

## Discussion

The computerized approach to a cancellation test implemented on a touchscreen and previously described in a pilot study [44] proved to be usefully applicable to a large population of control subjects and patients with unilateral brain lesions.

The observational study was performed on a population of adult subjects; 119 normal subjects as controls and 198 subjects with unilateral brain damage who were screened according a neglect testing battery. Apparently no subject with left brain lesion and neglect was identified.

The chosen task consisted of a visual searching involving cancellation of targets interspersed with distractors. This task is a widely adopted paradigm in clinical pencil-and-paper tests [4], [6]–[7] and, compared to other classical approaches such as line bisection or figures copying, is particularly fit for a quantitative instrumented approach. The proposed touchscreen approach allowed to analyze the spatial and temporal evolution of the searching performance, providing a set of indexes related to “how” the task has been fulfilled, in addition to the classic global score of “what” was the final result of the performance. Such additional indexes have already demonstrated their usefullness in classic pencil-and-paper tests [53]. The items' arrangement was shown on a 19-inch touchscreen monitor with a 4∶3 aspect ratio. The spatially pseudorandom distribution was preferred to the rows item arrangement because it is expected to be more challenging and it avoids a strong bias towards a reading-like search strategy which characterizes the items' arrangement on rows [44] and the display size was comparable with the apparent sizes of pencil-and-paper test sheets. The feedback provided on a touched target consisted of a tight circle around the item itself: this choice was preferred to crossing (often adopted in pencil-and-paper tests) because it better resembles a fingerprint or a pressed button and therefore it is assumed to be more ecological.

Among the study participants, those unable to detect at least 10 targets out of 40 (they were 5 right-brain-damaged patients with marked neglect) were excluded from the data analysis. The RUSN+ group is therefore intended to comprise patients without severe neglect, able to detect at least a quarter of presented targets.

### Rationales for indexes definitions

The proposed neglect score (NS) quantifies a right to left difference in the ability to recognize targets: in optimal performance NS is null, while increasing values mark an increasing unilateral spatial neglect (up to a maximum of 50%) where the sign marks the laterality (+ for right neglect, - for left neglect). It is worth remembering that, for classic pencil-and-paper neglect scores, the null value which characterizes optimal performance, can also be obtained in tests presenting the same number of omissions in both left and right hemifields.

The other proposed scores quantify the detected targets (NT), the touched distracters (ND) and the perseverations (NP). It should be noted that the finger-item contact algorithm is not affected by repeated contacts, possibly dueto tremor, and by finger sliding on the screen. The algorithm [44] classifies as perseveration any returning touch and any consecutive touch, given that a sufficiently large latency elapsed, though this latter occurrence is rare and substantially not observed in the present study.Accordingly, only involuntary screen-hand contacts may determine spurious touches possibly increasing the target or distracter score. When during the run-in trial such occurrences appear to be probable, the operator must provide enforced instructions to avoid such artefacts. Therefore, since this method is not applicable to patients with severe motor disturbances who cannot control involuntary hand-screen contacts, the risk of spurious touch in sufficiently able subject appears to be minimal, worth accepting against the provided advantages.

In order to provide a detailed description of “tactic” aspects of searching (i.e. how is negotiated the next target), a large set of indexes have been considered and a selection was performed discarding those indexes which were statistically related to others and thus avoiding a redundancy of information; the same check was performed by Manly [43] in order to identify the indexes which were independent from each other. Therefore, the proposed indexes included, in addition to global scores, a temporal index such as Latency (LI) and a spatial index such as Proximity (PI, the number of not yet cancelled targets closer to the previous one with respect to the one actually touched). While the Latency index shows a strict relation with the indexes involved in the chronometric approaches previously listed in the Introduction (while the related and discarded index SI is not as immediate and comparable with other studies), the Proximity index is innovative and its intent is to evidence how a search path does not necessarily consist of finding the closest target. This index is close to the Search Organization index proposed by Manly [43] which quantified how many times the search of a next target was “far” from the previous one (“far” meaning that the sectors of two consecutive target cancellations were not adjacent). On the contrary, the discarded related distance index DI [41] is strictly related to target arrangement.

Other indexes were intended to quantify the searching “strategy”: the direction indexes (XC and YC) quantifies the overall search direction, particularly showing large values when a clear search direction can be detected. The reader can find great similarities of these indexes with the “best r” proposed by Mark [41]: the mathematics behind are in fact the same and they also share an overall reference to a more or less organized search direction. However, in addition to the presence of a preferential search direction our couple of indexes also provides the actual direction. The identification of the first cancelled target may be considered an indicator that approximates the identification of the search direction [9], [43].

Another index, similar to an already proposed index [41] and related to the “level of organization” of the search strategy, is the count of actual path crossings (NC): those occurrences mark an inefficient, disorganized search path in which it may occur to rescan portion of the display already scanned. The related LP (Longest Path) index is believed to be less immediate than NC and therefore it was excluded. In any case all discarded indexes (DI, SS and LP) are not fully correlated with the retained ones, and therefore further analyses will possibly be carried out in order to evidence more specific information.

Finally, two indexes, the Latency Gradient (LG) and the Proximity Gradient (PG), are oriented to reveal a possible lateral gradient in the performance. Their definition introduces an advanced characterization of neglect: not only an on-off phenomena but also a gradual worsening of some aspects of the searching ability when moving the attentional focus towards one side (usually to the contralesional side). Therefore, the gradient indexes might identify a class of “mild” neglect subjects, showing normal cancellation scores along with a spatially unbalanced and impaired ability to search for targets.

These gradient indexes can be considered in close connection with published scientific works in which a gradient of the omission rate has been experimentally evidenced in neglect patients [18], [29], [30]–[31]. In those works, the display of a cancellation test was divided into 6 to 8 equal vertical columns, each characterized by a given omission rate which almost linearly increased moving controlesionally. Those results fit well with a “neglect gradient model” [54]–[55] in which it was hypothesized that visual attention, quantified by the amount of localized eye fixation, gradually decreases from the most ipsilesional field of view to the most contralesional one. This hypothesis has been experimentally confirmed by Behrmann [37], while evidences from other studies [39] did not support it. It must be noted that those latter studies were performed with large and different test displays which span respectively about 45° and 90°, while our setup provided stimuli under a 30° view angle, therefore it is arguable that the apparent differences were partly due to different display size [56].

### What is normal spatial exploration/search? Is the search performance of brain-damaged patients not normal?

The global scores obtained by controls in the touchscreen tests confirmed the expected figure where all scores are close to the ones associated with a “best” performance: all targets detected, no distracters cancelled and no perseverations observed. The performance indexes provided further details on “how” control subjects fulfill their task and, interestingly, the presented results demonstrated that a normal performance does not necessarily imply looking for the closest target available and avoiding rescanning of already searched areas, as quantified by path crossing. It is interesting to notice that a relevant part of those crossings took place in the final part of the exploration when the subject looks for the few targets still to be found. The task implicitly involves re-exploration and therefore produces path crossings. It is obvious how those path crossings have a different meaning from crossings that take place in the initial part of the performance [41].

It is also relevant to remark that in control subjects, performance indexes concerning time (latency) and space (proximity) showed no relation with the lateral coordinate, i.e. the same latency and same proximity indexes were observed all over the test display.

Finally the indexes concerning search strategy indicated that the large majority of controls scanned the display top-down with horizontal rasters. Fewer individuals scanned left to right with vertical rasters and a minority explored adopting other strategies, including some that did not show a preferential direction, such as a daisy-like pattern.

The results in the brain-damaged groups confirmed the general observation that brain lesions may alter the exploratory skill and search effectiveness. This was not only observed in the global scores, which in general confirmed the known outcomes of explorative performances in pencil-and-paper cancellation tasks (LUSN− and RUSN− groups showing occasionally a mild to moderate alteration of scores, and RUSN+ group showing large and relevant changes in those scores), but further details were provided by the additional indexes related to “how” the explorative task was fulfilled.

As to global score, the brain-damaged groups were differently characterized: the LUSN− patients particularly showed a slightly abnormal value for the contralesional neglect score (NS) and a large number of cancelled distractors (ND) and perseverations (NP); the RUSN− group, except for a neglect score which was slightly different from controls, showed an almost normal behavior, while the RUSN+ group, beyond an apparently large neglect score (NS), had significantly larger numbers concerning undetected targets (NT), distracters (ND) and perseverations (NP). Interestingly both LUSN− and RUSN− groups, though negative at the pencil-and-paper testing and able to cancel as many targets as the control subjects (normal NT scores), showed slightly abnormal neglect scores which is consistent with the basic notion that unilateral brain damage may alter the ability to explore the contralesional field [1]–[3].

All brain-damaged patients were slower (LI larger than normal) and, among them, particularly LUSN− and RUSN+ subjects. A slowing (negative LG) in the contralesional field was evidenced only in the right brain-damaged patients: the further to the left was the detected target, the slower was the RUSN+ group. The same outcome was also observed in the RUSN− group. Even if figures were lower than for the RUSN+ group, they were still significant.This latter evidence extends the conclusion “that the attentional deficit in neglect follows a left-right gradient” [37] also in subjects with right brain lesion but without neglect.

Proximity, i.e. an index related to the closeness of targets sequentially detected, was slightly but significantly larger in LUSN− and RUSN+ groups than controls, while in RUSN− it was not different from controls. The gradient index PG evidenced a significant decrease of proximity index in the contralesional field in LUSN− and RUSN+ groups. Such figure evidences that those two groups tended to detect targets closer to the previously touched ones on the contralesional visual field as compared to the ipsilesional field. As for the search path crossing (the NC index) all patients' groups showed larger values than controls: as already noted for latency and proximity indexes, the LUSN− group showed intermediate values between RUSN−and RUSN+ groups.

The RUSN+ group always showed the most abnormal values for the outcome scores and indexes, sometimes (as for ND, LI, PI, PG) sharing this primacy with the LUSN− group. Moreover, the RUSN− group generally showed values of scores and indexes which are intermediate between control (in 4 out of 9 indexes, RUSN− scored significantly different from controls) and RUSN+ groups (in 6 out of 9 indexes, RUSN− scored significantly different from RUSN+), thus evidencing that the occurrence of unilateral spatial neglect is a predominant factor in modulating all the different aspects characterising spatial visual exploration.

Nonetheless, other cognitive, visuoperceptual and motor factors may contribute to the abnormal scores of the brain-damaged groups: among others dexterity deficits [57], limited reliance on the able ipsilesional nondominant hand, disturbances related to language and reading such as alexia [58], misguided hand movement due to optic ataxia [59]. The specific balance of those factors certainly influences the individual performance and, when the association between a factor and the side of the brain lesion is known, some evidences can be interpreted accordingly: the worst ND and LI scores in LUSN− group may be related to their prevalent problems in the linguistic domain [58], particularly affecting the letter-based test, and/or to persisting long-term dexterity deficits in the ipsilesional upper limb after left hemisphere damage [57]. On the contrary, we are oriented in considering the PI differences across patient groups observed in the present study as a random effect: in fact, the index distribution is apparently very similar across groups and, after checking that no difference was observed between RUSN+ and RUSN− groups, a t-test comparison between pooled right brain-damaged groups and left brain-damaged group did not detect any difference. Further studies will be necessary to understand if abnormal PI is a nonspecific outcome of a brain damage or if it can be related to specific typology of patients.

This research indicates that the performance in a search test cannot be globally classified as normal or abnormal only on the basis of the final outcome, in this case a neglect score quantifying the unbalance between the number of omissions in the two hemifields, but other performance-related aspects concerning organization, velocity, efficiency should be taken into account. Accordingly an abnormal behaviour could be ultimately characterised by a normal neglect score associated with abnormal performance indexes: the differences in the search ability between the control group and the RUSN− group, which the touchscreen method had evidenced, demonstrates that an analytical approach, such as the touchscreen one, has a higher sensitivity, when compared to synthetic approaches such as the traditional pencil-and-paper tests, to factors potentially affecting the cognitive performance.

Conversely, the fact that 7 (out of 55) patients from the RUSN+ group had a normal neglect score NS at the touchscreen test does not contradict the stated higher sensitivity of the touchscreen method. First of all, it should be noted that even the comparison of scores of the five pencil-and-paper tests in the RUSN+ group (available in 50 out of 55 RUSN+ subjects) evidenced noticeable incongruencies: the majority (29 out 50) was neglect-positive just in one or two pencil-and-paper tests and only few individuals (2 out of 50) were neglect-positive in all five pencil-and-paper tests. Secondly, those 7 RUSN+ patients, who were positive at the pencil-and-paper battery and had a normal neglect score in the touchscreen test, had abnormal values in the other Performance Indexes measured by the touchscreen test.

### Search strategies and search tactics

It is useful to adopt the concepts of strategy and tactic in the analysis of a search task: tactic, literally the way a fighter negotiates a close encounter with an enemy, is in the present context how a subject negotiates the search for the next target, just after having cancelled one out. Strategy is an overall organized and recognizable evolution of the searching performance.

It is worth to note that strategy is a characteristic of a high level of intelligence, while the searching task could be fulfilled even in absence of a strategy and just adopting a working tactic (for example, always look for the closest available target). Typically, an exclusive tactical approach does not produce an efficient search performance (such as good soldiers cannot win without a good chief officer).

The XC/YC couple seems to represent well the search strategy: in this sense search strategy consists of a predefined project which stands throughout the entire task. As for the results, the most common search strategy among controls is to scan top-down horizontally the display. Along with a worsening of the search ability and neglect onset (higher NS) a search strategy in which patients scan leftward vertically the display is increasingly observed. This finding is in accordance to the one reported by Manly [43]: control subjects started in the upper left sector, while neglect patients started exploring on the extreme right sectors.

The presented results, which show values of indexes related to search strategic aspects NC, PI, XC and YC in the patients' groups that partially overlap the values observed in the control group, support the conclusions of previous studies in which a strict relation between neglect and chaotic search strategy was not always observed. Neglect patients can still perform an organized search [41] and, accordingly, a strong association was not found in RUSN+ patients between neglect score NS and organization-related index NC (r_NS,NC_ = 0.47).

Other indexes, such as Latency (LI) and Proximity (PI), seem more related to tactical aspects. It is possible to hypothesize a set of alternative target tracking tactics: a Closer target tactic characterized by extremely low PI values (for null PI values, it becomes a Closest target tactic in which the next searched target is the closest one); a Miner tactic when it is preferred to maintain a search direction approximately in front of the current direction; a Climber tactic (in which the subject explores following an imaginary winding lane); a Chaotic motion (the perfectly disorganized approach in which any target tracking restarts from a full display analysis and produces a search path that jumps from any target couple). Every single one of these tactics is compatible with any organized search strategy (with the exclusion of the chaotic one). It is worth noting that Proximity tended to decrease in the contralesional visual field in RUSN+ and LUSN− groups: such evidence could be interpreted as resulting from a more tactical (or, alternatively, a less strategical) approach in the contralesional field.

### Conclusions

The proposed testing setup is able to provide detailed insight in the human visual exploratory skill.

Normal control subjects show variability of indexes displaying how it is not possible to identify one single normal modality. This evidence may be related to the redundancy of the cognitive resources in normal subjects.

Despite controls' variability, groups of homogeneous patients with unilateral brain lesion (right or left) and possible cognitive disturbances (neglect absent or present) show a statistically significant difference in almost any index considered. Also, differences are evidenced within each group.

The results demonstrate that right brain-damaged patients not showing neglect at pencil-and-paper tests (RUSN−) are positioned between controls and neglect patients. They seem to belong to a “gray” area where neglect, as it is commonly defined, is not present but nonetheless the exploratory skills cannot be assumed as “normal”.

Particularly the gradient indexes have showed how this mild exploratory disorder is strictly connected to neglect: while neglect is the inability to identify targets in some part of the visual field (typically left), these subjects show increasing difficulties and worsening performances when searching target towards the left visual field. The gradient of search performance indexes is nonetheless often present in the ipsilesional side of the display.

**Figure 9:**
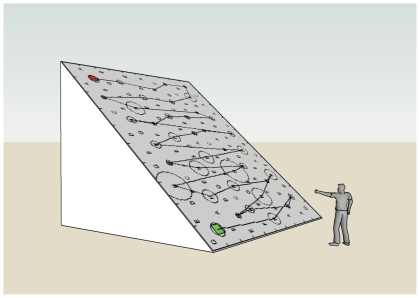
The occurrence of a raster-like path perpendicular to the Search Direction in a cancellation task can be figured out as an efficient path trajectory in an uphill search exploration task, where the hill slope represents the intrinsic difficulty in exploring the contralesional field.

Confirming previous remarks from the literature [41] it has also been observed that neglect does not necessarily imply a disorganized search: normal subjects tend to prefer a top down search strategy alternating horizontal rasters from left to right and right to left. Neglect patients and also a part of right brain-damaged patients without clinical neglect tend to prefer, when able to perform an organized search, a right to left search strategy alternating vertical rasters. While a discussion of the reasons about the normal preference for a reading-like strategy is beyond the scope of this paper, the right brain-damaged patients preferred strategies in line with the gradient concept. If we consider that the subject is aware of his difficulty of exploring towards left, we can imagine his exploratory task such as tracking mushrooms on a mountain slope. The subject planning an efficient search strategy will also try to minimize his cost and an obvious result is to explore the mountain slope following a path that winds up the slope exploring one strip of terrain and then moving to the upper strip (see figure 9). In this sense the least efficient strategy would have been to continue to go up and down the slope. Such a hypothesis may help in adding a strategic element into mathematical models simulating visual search [60]. In fact, the cited model appears to simulate well a visual search of the most impaired neglect patients, who besides a clear negligence of the left space, are characterized also by a somewhat chaotic search path, typical of a tactical approach which misses a strategic project. On the other hand, the proposed model seems unable to simulate a plausible normal organized search path because of the apparent absence of a search strategy.

In conclusion, while the present study and related results are referred to homogeneous groups, the potential impact of the proposed methods on the assessment of an individual in a clinical context has to be considered according the following final considerations:

- the large experimental campaign demonstrated the compatibility of the touchscreen setup with a clinical context, thus fostering new developments, already activated, in testing tablet-based setups possibly supporting tests to be administered even at the patient bed in acute stroke units in order to profile the evolution of cognitive functions following focal brain damage;
- while the traditional pencil-and-paper tests can identify a cognitive deficit only when targets are omitted in a search and cancellation task, the proposed touchscreen-based test can provide a detailed insight in the spatial exploration function, enabling identification of abnormal behavior also when omissions do not occur. The abnormal features of the RUSN− group performances, evidenced by the experimental results, demonstrated the higher sensitivity to abnormal behaviors of an analytical approach, such as the proposed touchscreen-based one;
- the proposed indexes quantify both tactical functional aspects such as those involved in the “next” target detection and strategic functional ones such as features concerning the search-path. Moreover, the results demonstrated that those indexes are quite independent and therefore quantify different aspects of the cognitive ability in exploration tasks; such analytical assessment may support and possibly improve the clinical decision making about therapies and rehabilitation programs [45]. Such clinical potential should be necessarily supported by future test-retest reliability studies.
