# Correction: Spatio-Temporal Features of Visual Exploration in Unilaterally Brain-Damaged Subjects with or without Neglect: Results from a Touchscreen Test

**DOI:** 10.1371/annotation/bf311a56-bc48-44b6-9b0f-7ccc97fc290f

**Published:** 2012-03-29

**Authors:** Marco Rabuffetti, Elisabetta Farina, Margherita Alberoni, Daniele Pellegatta, Ildebrando Appollonio, Paola Affanni, Marco Forni, Maurizio Ferrarin

There was an error in the first affiliation. The correct first affiliation is 1 Fondazione Don Carlo Gnocchi ONLUS, Italy. 

